# Relationship between age, BMI, head posture and superficial neck muscle stiffness and elasticity in adult women

**DOI:** 10.1038/s41598-019-44837-5

**Published:** 2019-06-11

**Authors:** Piotr Kocur, Maciej Tomczak, Marzena Wiernicka, Magdalena Goliwąs, Jacek Lewandowski, Dawid Łochyński

**Affiliations:** 1 Poznan University of Physical Education, Department of Musculoskeletal Rehabilitation, Poznan, Poland; 2 Poznan University of Physical Education, Department of Psychology, Poznan, Poland

**Keywords:** Predictive markers, Skeletal muscle

## Abstract

This study determined relationships between age, BMI and cranio-vertebral angle (CVA) (independent variables) and stiffness and elasticity of sternocleidomasteoid [SCM] and upper trapezius [UT] (dependent variables) muscles in sitting posture in 95 women across adult life. Moreover, a stepwise regression was performed to determine to what extent the dependent variables are explained by age, BMI and CVA. Age was moderately correlated with BMI (r = 0.41), and both age and BMI were moderately negatively correlated with CVA (r = −0.54 and −0.55, respectively). High (r = 0.73) and moderate (r = 0.53) linear relationships were present between age and logarithmic decrement (inversely related to elasticity) and stiffness of SCM muscle, respectively. Low (r = 0.36) and moderate (r = 0.47) relationships were present between age and logarithmic decrement and stiffness of UT muscle, respectively. Age accounted for 53% variance in elasticity and 28.5% variance in stiffness of SCM, and for 13% variance in elasticity and 22% variance in stiffness of UT muscle. Introduction of BMI but not CVA to the model explained the variance of these parameters by additional 0–8%. Among the studied factors age is the major correlate of stiffness and elasticity of neck muscles across the adult life.

## Introduction

Aging results in progressive changes in the structure and function of the neuromuscular system that limits physical performance, and leads to disability, decreased autonomy, and quality of life^1^.

Skin^2^, subcutaneous^3^, myofascial^4^, and skeletal muscle tissue undergoes substantial remodelling^5^ with aging. Various structural and biochemical changes to cellular and extracellular matrix of muscle tendon unit have been reported in humans and rodents, encompassing muscle fiber type redistribution^6^, alterations in connective tissue protein concentration and cross-linking^7^, as well as structural degeneration^8^. In humans the above-mentioned morphological and structural adaptations were mainly studied in the lower and upper limbs^9,10^. However, Mezranic *et al*.^11^ also found noticeable changes in fiber structure and morphology in neck muscles, such as sternocleidomasteoid muscle [SCM].

With aging the degenerative changes in the connective tissue lead to decrease in elasticity of integumentary^2,12^ and musculoskeletal system^8,13^, and to increase in muscle stiffness^14^. Elasticity is the mechanical property which describes an ability of a body to recover its previous configuration, after it was deformed by an applied load. The higher the muscle/myofascial elasticity, the greater the body ability to spring back to its original shape^15^. Stiffness is the resistance of the body to an external force that deforms its initial shape^16,17^. Myofascial stiffness is one of the essential indicators of energy storage of muscle-tendon unit, and can affect control of joint motion. In recent years, the biomechanical parameters reflecting adaptive changes in viscoelastic and mechanical properties of various skeletal muscles have been increasingly studied. Up to date significant differences in muscle stiffness parameters were found between the elderly and young individuals using sonoelastography^18^ or MRI^19^. Furthermore, using myotonometry, a reliable and reproducible non-invasive measurement method, higher stiffness values and lower elasticity of superficial muscles of neck^20^ and face^21^ were found in old individuals compared to young ones. However, it is not known how these mechanical properties of neck muscles change between third and ninth decade of life, and whether they are affected by other factors such as body fat or head posture.

Gradual decrease in muscle mass (atrophy) has been shown in humans, starting from the 3rd decade of life^22^, and is particularly noticeable in the 5th decade of life^23^. Loss of muscle strength and power (dynapenia) has been also recognized as a serious debilitating condition that leads to life threatening physical failure^10,22,24^. Decline in strength can lead to poor posture, which is one of the most observable structural change in the upper part of the body with age. Shoulders become rounded while thoracic spine kyphotic^25^. The cervical lordosis is increased, and the neck mobility is reduced^26,27^. Head becomes anteriorly positioned, which leads to forward head posture (FHP). It has been shown that anterior positioning of the head increases linearly with age^28^.

With aging extracellular matrix becomes infiltrated with fat, which is an another structural change in the skeletal muscle, known as myosteatosis^29^. Aging is associated with changes in body composition and fluctuations of BMI. In the last decades of life, there is a visible increase in adipose tissue and decrease in lean body mass, especially in muscle tissue^30^.

The aim of the study was to determine the relationship between age, BMI, and position of head, and stiffness and elasticity of the SCM and upper trapezius (UT) muscles in adult women. These superficial neck muscles have been chosen since they demonstrate lower susceptibility to atrophy and relatively unchanged pattern of activity across life compared to limb muscles^23^. Thus they may well reflect alterations in mechanical myofascial properties during healthy aging^11^. So far, no attempt has been made to determine changes in biomechanical parameters of superficial neck muscles with aging, while their alterations could become an important predictor of biological age. As BMI, forward head posture and muscle stiffness have been shown to be increased and muscle elasticity to be decreased in old individuals, we hypothesized that this process would start in the early adulthood and continue with age. We also tested the hypothesis that age will be the strongest predictor affecting neck muscle mechanical parameters.

## Material and Methods

### Participants

The study involved 95 volunteers at various stages of life who met the inclusion criteria. Only healthy women over the 20 years of age, without pain in the cervical spine and neck over the last 6 months (VAS <3, NDI <8%/4 points), who did not undertake any regular physical activity during the last year were recruited. Only women were recruited, as they have potentially different distribution of adipose tissue in the muscle system than men^31,32^, as well as potentially different composition of muscle fibres^20^. Exclusion criteria were as follows: (1) neurological deficits, (2) pathological musculoskeletal dysfunctions i.e. spinal deformities such as torticollis, scoliosis (visible rib hump), osteoporosis, or visible kyphotic deformities of the spine being a consequence of serious disease such as Scheuermann disease, Gibbus deformity or ankylosing spondylitis, (3) acute and chronic pain syndromes in the region of the neck and shoulders, (4) previous surgical procedures in the region of the chest, shoulder girdle and cervical spine. We also excluded women taking myorelaxants and other drugs that may affect the muscle tissue properties. One hundred sixty nine participants meeting the inclusion criteria responded to the study announcement. After completing the questionnaire and conducting an interview, 74 women who did not meet the inclusion criteria were excluded.

The participants were sought among students, employees of universities, private computing centres and corporations as well as individuals from senior care homes. Participants were recruited on the basis of questionnaire in which subjective assessment of health and leisure time of physical activity levels were included according to The Stanford Brief Activity Survey^33^. The Polish version of Neck Disability Index Questionnaire^34^, and intensity of the current pain in the neck area using a visual analog scale (VAS) were assessed.

The Institutional Review Board of the Poznan University of Medical Sciences approved the study. The experimental procedures were conducted in conformity with the Declaration of Helsinki. The relevant guidelines and regulations of the local institute were strictly followed when conducting the study. Each participant signed an informed consent form prior to participation.

### Procedure

**Figure 1:**
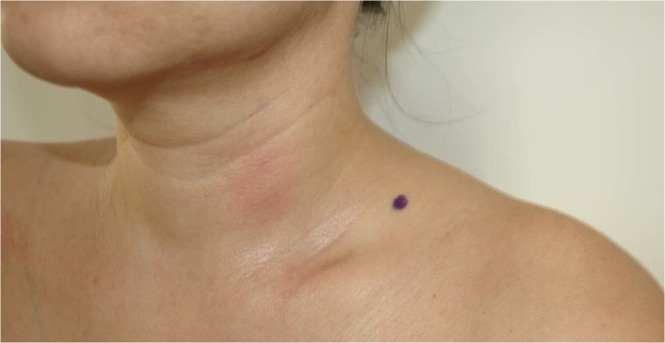
MyotonPro measurement point on the UT muscle.

**Figure 2:**
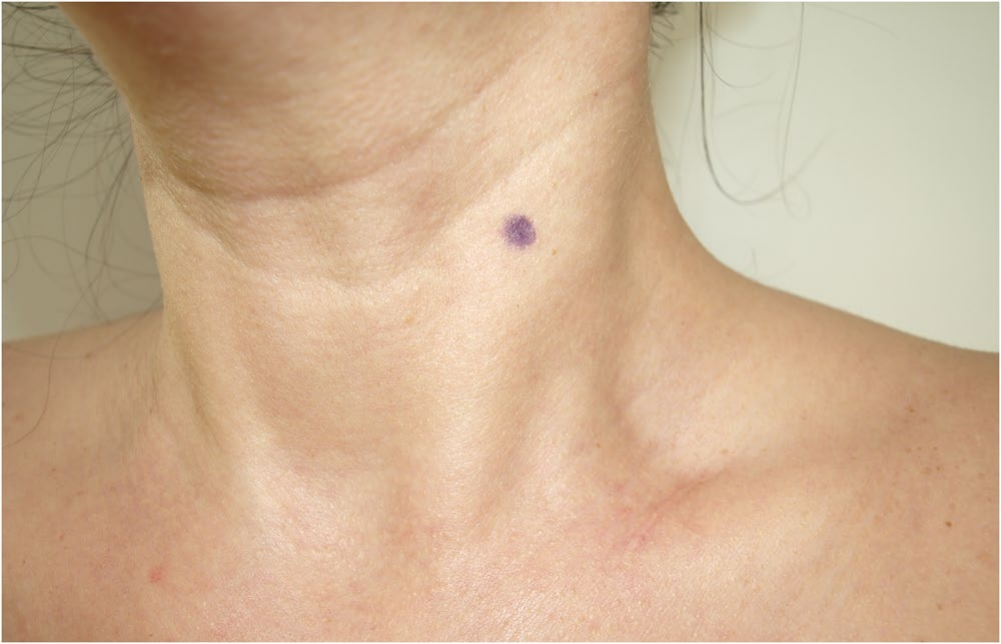
MyotonPro measurement point on the SCM muscle.

After collecting anthropometric measures, the measurements of myofascial tissue stiffness and elasticity were made using the MyotonPro® device (Myoton AS, Tallinn, Estonia). The tests were conducted in a sitting position on a chair with hands placed over knees. The subjects were asked to adopt a comfortable sitting position, typical for everyday activities, and to focus their eyes on the face-to-face screen for a few minutes. Initially, UT and then SCM muscle was examined. The measurements were performed first on the right, and then on the left side of the body. The UT was examined in the area of the cervical triangle, in the front of the muscle, on the line connecting the acromion and C7 spinous process (Fig. 1). The SCM was examined at a point located midway between the insertion to the anterior surface of manubrium sterni and the mastoid process of the temporal bone, in a place where both muscles heads are connected (Fig. 2). Each time, the probe (3 mm diameter) of the device was placed perpendicular to the surface of skin with constant preload (0.18 N). Measurements of mechanical muscle parameters were performed once, by the same assessor. The evaluation and measurements took place in a separate bright rooms (temp. 18–22 °C), in the mid of the week in the morning.

### Measuring of craniovertebral angle

The CVA was determined using a photometric method based on the standards described in the available literature. Generally, measuring of CVA in standing position is considered as a more sensitive method of FHP estimation than in sitting position^35^. Thus, the participants were examined in a standing position. Before taking the image, the participant was asked to fully flex and extend the cervical spine. Then, the C7 and tragus were marked. The camera was placed 1.5 m from the lateral surface of the body at the level of the acromial process. The upper part of the body was photographed from the side. After taking images, CVA (°) was measured as the angle between the line connecting the seventh cervical vertebra and the tragus of the right ear, and the line running horizontally through the C7 spinous process. The smaller the CVA denotes the larger FHP.

### Dependent variables

**Figure 3:**
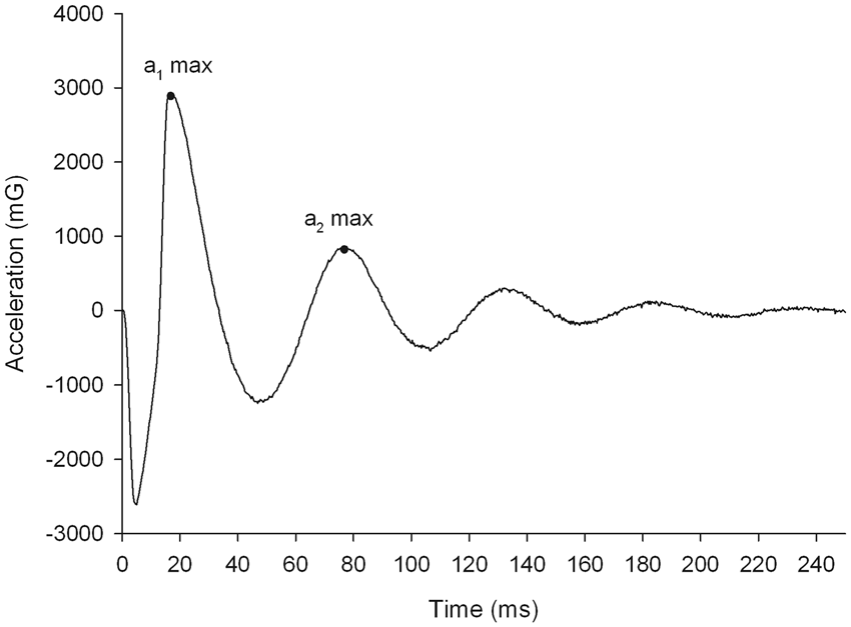
Example of raw acceleration signal from the upper trapezius muscle of one participant obtained with MyotonPro.

Muscle stiffness [N/m] was expressed as resistance of tissue to an external mechanical impulse^17^. The myofascial tissue oscillations were evoked with 10 brief (15 ms) mechanical impulses at 0.4 N force and frequency of 1 Hz. The dynamic myofascial stiffness (N/m) has been calculated according to the following manufacturer’s formula^36^ (1):

1 $$S={{\rm{a}}}_{1{\rm{\max }}}\cdot \frac{{m}_{probe}}{{\rm{\Delta }}l}$$

where: *S* – the dynamic stiffness; a_1max_ – the maximum acceleration where the equilibrium between the impulse force and tissue resistance is achieved (the point in time - Fig. 3 - where the maximum tissue displacement is achieved), m_probe_ – mass of the probe (preload 0,018 kg), Δl – the maximum tissue displacement amplitude (calculated automatically based on the mathematical algorithms created by the manufacturer of the apparatus).

In addition, muscle elasticity described as an ability to restore its superficial shape after being deformed was measured. Elasticity was calculated according to the manufacture’s formula^36^ (2) as the magnitude of logarithmic decrement (expressed in arbitrary units) in the amplitude of the second natural tissue oscillation in relation to the first oscillation evoked in response to the single, external mechanical impulse:

2 $$D=In(\frac{{a}_{1max}}{{a}_{2max}})$$

where: *D* – the logarithmic decrement; a_1max_ – the maximum acceleration where the equilibrium between the impulse force and tissue resistance is achieved (the point in time where the maximum tissue displacement is achieved); a_2max_ – maximum acceleration of the second period of oscillation which takes place due to the recuperation of stored residual mechanical energy in the tissue (Fig. 3). The logarithmic decrement is inversely proportional with the elasticity. Therefore, if the decrement decreases, the muscle elasticity increases.

### Statistical analyses

To assess the distribution of the data, the Kolmogorov–Smirnov test was performed. The calculated values of mechanical parameters (ten successive 15 ms mechanical impulses performed at 1 Hz) were averaged for each side of the body and then, again averaged for both sides. Pearson’s correlation coefficient (r) was used to assess the strength of relationships between age, BMI, CVA (independent variables), and stiffness and elasticity (dependent variables) of the SCM and UT muscles. The stepwise multiple linear regression with forward selection was conducted to test the influence of independent variables on the variance of neck muscle’s elasticity and stiffness. Only the independent factors that correlated significantly with the dependent variables during the multiple regression analysis were included in the model. The critical level of significance was set at α = 0.05.

## Results

In the Table 1, the demographics of all studied individuals were presented.

**Table 1:** Basic characteristics of participants (n = 95).

Parameters	Mean ± SD	Min-Max
Age (years)	48.8 ± 18.7	21–88
BMI	24.6 ± 3.7	18.5–33.8
Mass (kg)	67.7 ± 15.1	44–100
Height (cm)	165.3 ± 9.1	148–183
CVA (°)	44.4 ± 6.9	29–59
NDI score level (%)	4.8 ± 2.5	0–8
VAS (point)	1.0 ± 0.5	0–2

BMI - body mass index, CVA - craniovertebral angle, NDI - neck disability index, VAS - visual analogue scale.

### Relationship between age, BMI and CVA

Age was moderately positively correlated with BMI (Table 2). Both age and BMI were moderately negatively correlated with CVA (Table 2). With more advanced age and larger BMI values subjects had smaller CVA values, that is greater anterior positioning of the head. In the overall multiple regression model BMI and age explained about 41% of variance in CVA. There was a statistically significant negative relationship between age and CVA (β = −0.38, p < 0.001) and statistically significant negative relation between BMI and CVA (β = −0.40, p < 0.001).

**Table 2:** Relationship between age, BMI and craniovertebral angle and elasticity and stiffness of upper trapezius and sternocleidomastoid muscles (Pearson’s correlation coefficient r values) (n = 95).

	UT elasticity (log. decr.)	SCM elasticity (log. decr.)	UT stiffness (N/m)	SCM stiffness (N/m)	Age (yrs)	BMI	CV angle (°)
Age (yrs)	r = 0.36 p < 0.001	r = 0.73 p < 0.001	r = 0.47 p < 0.001	r = 0.53 p < 0.001		r = 0.41 p < 0.001	r = −0.54 p < 0.001
BMI	r = 0.33 p < 0.001	r = 0.38, p < 0.001	r = 0.44 p < 0.001	r = 0.45 p < 0.001	r = 0.41 p < 0.001		R = −0.55 p < 0.001
CVA (°)	r = −0.30 p < 0.05	r = −0.47, p < 0.001	r = −0.26 p < 0.05	r = −0.40 p < 0.001	r = −0.54 p < 0.001	r = −0.55 p < 0.001	

SCM - sternocleidomasteoid, UT - upper trapezius, BMI - body mass index, CVA - craniovertebral angle. Correlations are significant at p < 0.05.

### Elasticity of upper trapezius and sternocleidomastoid muscles

**Figure 4:**
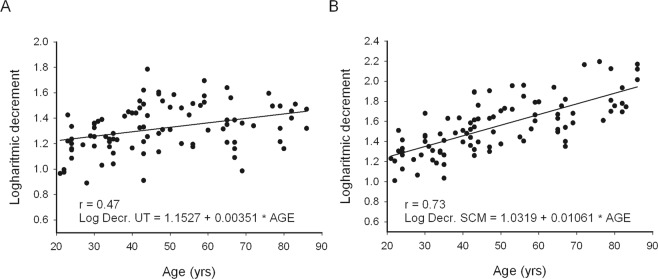
Elasticity (log. decr.) of UT (**A**) and SCM (**B**) muscles.

Logarithmic decrement (inversely related to elasticity) of neck muscles was positively correlated with age (Fig. 4). The relationship was strong for the SCM and moderate for the UT (Table 2). Also, BMI was positively related with elasticity of both muscles (Table 2). The strength of relationship was moderate and similar for both muscles. Hence, with more advanced age and larger BMI values subjects had lower elasticity in the superficial neck muscles. CVA was weakly negatively correlated with logarithmic decrement in both studied superficial neck muscles (Table 2). It means that greater anterior positioning of the head coincided with lower elasticity of neck muscles.

For the UT muscle, the results of the stepwise multiple regression analysis indicated that there were positive relations between age and elasticity and BMI and elasticity (Table 3). In the first step, age explained about 13% of the variance while BMI introduced into the second step explained additionally by about 4% of variance in elasticity. Age and BMI explained in total about 17% of the UT elasticity variance (R^2^ adj. = 0.15) (Table 3). The relationship between the elasticity of the UT, and the CVA introduced in third step in regression model, was not statistically significant.

**Table 3:** Regression analysis predicting elasticity of UT in sitting position (n = 95).

Variable		β	t	p	F, p	R^2^ add.	R^2^	change R^2^
Step 1	Age	0.36	3.68	0.000	13.57, <0.001	0.118	0.127	0.127
Step 2	Age	0.27	2.56	0.012	9.24, <0.001	0.149	0.167	0.040
	BMI	0.22	2.10	0.038				
Step 3	Age	0.24	2.13	0.036	6.19, <0.001	0.142	0.169	0.002
	BMI	0.19	1.68	0.096				
	CVA	−0.06	−0.49	0.626				

SCM - sternocleidomasteoid, UT - upper trapezius, CVA - cranio-vertebral angle, BMI-body mass index.

In case of the SCM, only the positive relationship between age and muscle elasticity was found in the first step, which explained about 53% of the variance (Table 4). The relationships between elasticity of the SCM, and CVA introduced in the second step, and the BMI introduced in the third step and in regression model, were not statistically significant.

**Table 4:** Regression analysis predicting elasticity of SCM in sitting position (n = 95).

Variable		β	t	p	F, p	R^2^ add.	R^2^	change R^2^
Step 1	Age	0.73	10.27	0.000	105.44, <0.001	0.526	0.531	0.531
Step 2	Age	0.67	7.98	0.000	54.02, <0.001	0.530	0.540	0.009
	CVA	−0.11	−1.32	0.189				
Step 3	Age	0.66	7.73	0.000	36.06, <0.001	0.528	0.543	0.003
	CVA	−0.08	−0.86	0.391				
	BMI	0.07	0.77	0.440				

SCM - sternocleidomasteoid, UT - upper trapezius, CVA - cranio-vertebral angle, BMI-body mass index.

### Stiffness of sternocleidomastoid and upper trapezius muscles

**Figure 5:**
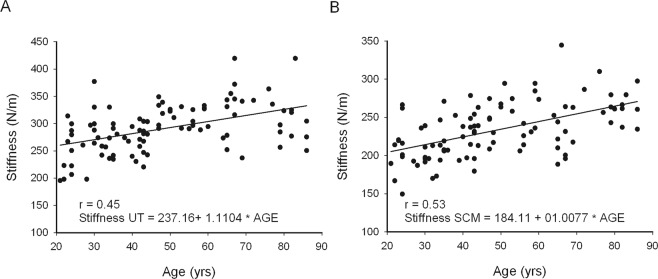
Stiffness of UT (**A**) and SCM (**B**) muscles.

Moderately positive linear relationships were present between age as well as BMI and stiffness of the SCM and UT muscles (Table 2). Advanced age (Fig. 5) and higher BMI values coincided with greater stiffness of neck muscles. Weak and moderate negative relationship was present between CVA and the SCM and UT stiffness, respectively (Table 2). This means that greater anterior positioning of the head coincided with higher stiffness of the superficial neck muscles.

In case of the UT muscle, the results of the stepwise multiple regression analysis indicated that there were positive relations between age and stiffness, and BMI and stiffness (Table 5). Age alone accounted for 22% of variance of the UT stiffness, while introduction of BMI in the second step to the model explained the variance approximately by additional 7.5% (Table 5). Age and BMI together accounted for about 29.5% of the variance in stiffness (R^2^ adj. = 0.28). The relationship between stiffness of UT, and the CVA introduced in third step was not statistically significant

**Table 5:** Regression analysis predicting stiffness of UT in sitting position (n = 95).

Variable		β	t	p	F, p	R^2^ add.	R^2^	change R^2^
Step 1	Age	0.47	5.10	0.000	25.98, <0.001	0.210	0.218	0.218
Step 2	Age	0.34	3.58	0.001	19.20, <0.001	0.279	0.294	0.076
	BMI	0.30	3.15	0.002				
Step 3	Age	0.40	3.84	0.000	13.54, <0.001	0.286	0.309	0.014
	BMI	0.36	3.45	0.001				
	CVA	0.16	1.37	0.175				

SCM - sternocleidomasteoid, UT - upper trapezius, CVA - cranio-vertebral angle, BMI-body mass index.

For the SCM muscle multiple regression analysis indicated that there were positive relations between age and stiffness, and BMI and stiffness (Table 6). In the first step age explained about 28.4%, while BMI introduced in the second step explained additionally about 6.4% of the variance (Table 6). Age and BMI together accounted for about 35% of the variance in stiffness (R^2^ adj. = 0.33). The relationship between the stiffness of SCM, and the CVA introduced in third step was not statistically significant.

**Table 6:** Regression analysis predicting stiffness of SCM in sitting position (n = 95).

Variable		β	t	p	F, p	R^2^ add.	R^2^	change R^2^
Step 1	Age	0.53	6.07	0.000	36.89, <0.001	0.276	0.284	0.284
Step 2	Age	0.42	4.55	0.000	24.59, <0.001	0.334	0.348	0.064
	BMI	0.28	3.01	0.003				
Step 3	Age	0.41	4.00	0.000	16.26, <0.001	0.328	0.349	0.001
	BMI	0.26	2.58	0.011				
	CVA	−0.03	−0.31	0.761				

SCM - sternocleidomasteoid, UT - upper trapezius, CVA - cranio-vertebral angle, BMI-body mass index.

### Changes in stiffness of sternocleidomastoid and upper trapezius muscles across the adulthood

The slopes of lines calculated from regression equations reflecting increase in stiffness and decrease in elasticity ranged from 1.49 to 1.52% per year between the third and ninth decade of life (Figs 3 and 4) for both studied muscles.

## Discusion

The major finding of the study is that there is a continuous increase in BMI and anterior positioning of head in women throughout the adulthood, which coincide with decrease in elasticity and increase in stiffness of the superficial neck muscles in sitting posture. However, age is the major correlate of stiffness and elasticity of superficial neck muscles. The predictive value of BMI in differentiation of muscle stiffness and elasticity is low, while anterior positioning of the head does not contribute to the variance of mechanical parameters of the studied muscles. Probably, this is because both these factors are mutually related with age. Furthermore, although age and to some extent BMI accounts predominantly for the variance in stiffness and elasticity of studied muscles, still large percentage of variance in myofascial mechanical parameters is left unexplained. This suggest that some other factors contribute to the differences in stiffness and elasticity of the superficial neck muscles in sitting posture. It should be noted that electromyographic activity of neck muscles was not monitored in this study, what would be helpful in understanding what accounts for the rest of variance in measured parameters. Therefore, it is not known, how substantial was the contribution of passive elastic and contractile stiffness to the measured muscle properties^15^.

### Age and mechanical properties

In previous studies performed with the use of myotonometery, higher stiffness and lower elasticity values were found in the biceps brachii and rectus femoris^37^, as well as the SCM and UT muscles^20^ in old as compared to young individuals. Also, higher values of stiffness of neck and face muscles^21^ were reported in older people. As previously observed, we found that although the SCM had lower stiffness and higher elasticity than the UT muscle^20^, the pace of change in both mechanical parameters studied (i.e. decrease in elasticity and increase in stiffness) was very similar during the adulthood. On the other hand, mechanical parameters of both muscles were affected differently by aging. We noticed, that age as a predictor explained the variability in stiffness and elasticity parameters more significantly in the SCM than UT muscle. With aging the antigravity function of superficial neck muscles increases at the expense of decrease of the deep neck flexors muscle function^38^. However, while increase in electrical activity as well as stiffness of UT muscle in sitting position is well documented, previous EMG studies have shown that SCM is quite relaxed in sitting position^39^, and its electrical activity is not depended on head posture^40^. Simultaneously, biomechanical parameters (stiffness, elasticity) of SCM are not changed during transition from lying to sitting position, regardless of age^20^. Therefore, mechanical parameters of SCM are presumably influenced more by passive structural properties, while for UT muscle these characteristics might be masked, as it is under active state of tension during sitting. This fact may indicate that mechanical parameters of the SCM better predicts biological aging as compared to the UT, and can serve as a simple muscular biomarker to evaluate muscle health with aging. This is in line with the work of Mezarnic *et al*.^11^, who showed that the size of the SCM muscle fibers is maintained relatively unaltered across the life. However, before mechanical properties of the SCM can be taken into account as the marker of aging, greater number of subjects, including children and men, need to be studied in the future.

The explanation of the mechanism affecting elasticity and stiffness in the superficial neck muscles with age is not obvious. Myotonometric measurements of muscle biomechanical properties depend on many variables related to the structure and function of the connective tissue. This tissue creates the layer composed of superficial and deep fascia, which surrounds and penetrates muscles tissue. Structure and morphology of myofascial tissue change constantly and dynamically in response to external loads^41^. It is known that extracellular matrix morphology, i.e. content of collagen and elastin^42^, and particularly collagen cross-linking^43^, can influence stiffness and elasticity. Therefore, we suppose that the most probable reason for an increase in stiffness and decrease in elasticity is muscle connective tissue infiltration and degeneration. It has been shown in various studies that the amount of non-twitching-connective or adipose tissue in skin and myofascial tissues increases with age, which affects both higher passive muscle stiffness, passive elastic properties, as well as quality of muscle contraction^2,8,12–14,42–44^. It seems reasonable to conduct further more detailed laboratory histological or imaging studies of the SCM and UT muscles to determine not only the amount, but also the composition of the myofascial tissue. This would help to understand the mechanisms behind alterations of stiffness and elasticity of studied muscles with aging.

During muscle contraction, muscle fibres transmit the force to the surrounding connective tissue, that is endomysium, perimysium and epimysium^44^. Thus, changes in the quantity and quality of fibres in the muscles^43,45–47^ might also affect structure and biomechanical properties of neck myofascial tissues. For instance, changes in muscle fibre proportions towards the slow phenotype^11,46^ could cause decrease in elasticity and increase in stiffness of neck muscles across lifespan. In the elderly, atrophy affects predominantly type 2 fast muscle fibres^9,22^, and causes that old muscles are composed of predominantly slow fibres, which seems to be stiffer than fast muscle fibres^42^.

### BMI and mechanical properties

Even though BMI is the strongest predictor of mortality among other anthropometric indicators^48^, in our study BMI moderately correlated with age, and only slightly explained the variance in stiffness [7.5% for the SCM and 6.5% for the UT] and elasticity [4% for the UT] of studied muscles. Previous studies using sonoelastography indicated that BMI is an additional factor that correlates with passive muscle stiffness of the UT muscle^49^. However, this was not confirmed by subsequent reports. Inclusion of BMI as a covariant did not affect the biomechanical muscle parameters evaluated using different measurement methods^18,20^. Therefore, the influence of adipose tissue content on muscle elasticity and stiffness is ambiguous. It seems that the inclusion of the BMI as an indicator of body fat composition, which may explain changes of the elasticity or stiffness of skeletal muscles across the lifespan, requires a more detailed consideration. For example, possibly decrease in height due to change in body posture causes only apparent increase of BMI with aging. Moreover, loss of muscle mass, increase in connective tissue content and change in distribution of body fat with aging is not expressed within the BMI^50^. Thus, the use of a classic BMI index calculated from height and weight might be inappropriate method for estimation of body composition with aging, which introduces bias in estimation of contribution of body composition to myofascial stiffness with aging. Therefore, we believe that other body composition measures, such as fat mass index, fat free mass index, estimation of lean body mass or even subcutaneous fat thickness should be considered as factors, which can explain changes in stiffness and elasticity with aging.

### CVA and mechanical properties

Hypothetically, the biomechanical properties of the muscles in the neck region of the body may to a certain extent be dependent on reduced CVA angle with aging. Although we noticed a moderately strong negative correlation of CVA with age, which confirms previous conclusions about the possible correlation of FHP with age^28^, anterior positioning of the head did not account at all for the variance of mechanical properties of the superficial neck muscles. This is in line with the reports showing that FHP does not influence neck muscle morphometry and activation^51,52^ nor electromyographic activity during maintaining head posture in sitting^40^.

### Practical consideration and future research

The important practical aspect of our study is that the elasticity of the SCM, measured at the specific location of over the muscle belly, can become a biomarker for physical state of skeletal muscles^11,53,54^, and may serve in the future as a simple estimator of the body biological age. Moreover, although the degenerative changes in the myofascial tissue are irreversible and progress with time, the effects of aging can be delayed^43,55^, and muscle function improved. The passive elastic stiffness of very inactive and sedentary people may be different from the active women^56^. It is known that a minimal level of physical activity may prevent the accumulation of connective tissue in the aged muscles^56^. In future, assessment of stiffness and elasticity of the SCM and UT muscles could provide important knowledge for evaluation and programming of specific therapeutic interventions directed to improve muscle function in the neck region.

Although, among studied factors predominantly age accounted for all the variability in stiffness and elasticity, still large percentage of variance of these mechanical parameters was left unexplained. This suggest that other factors such as level of neuromuscular activity, the current function and load of the superficial neck muscles and structural composition of myofascial tissue perhaps contribute to differences in mechanical parameters of the superficial neck muscles in sitting posture. Differences in stiffness and elasticity among individuals can result from differences in shape and structure of neck muscles and in isometric tension used to maintain head in space^41^, which probably arise from anatomical differences or habitual activity of neck muscles during daily life activities. Further studies on physiological properties of the SCM and UT seems justified, since the changes in this region are not only the reason for mobility restriction in the cervical region^57^, but also may cause musculoskeletal imbalance^58^ in the elderly.

## Study Limitation

Although our study provides information that may be important in assessing the processes of muscular aging, there are some limitations that do not allow unambiguous conclusions for the entire human population. First of all, we decided to examine only a group of women. Nevertheless, both muscle strength, and the content and distribution of adipose tissue of men and women are different^31,32^. In addition, the muscles of women and men have a potentially different composition of muscle fibres^23^, and this can affect the interpretation of the results. Therefore, we decided to choose a homogeneous research group composed of only women. The second limitation is that examination of muscles was performed only in a sitting position, which can limit the inference about the passive properties of the muscles. However, by including the measurement of the CV angle as a potential factor affecting the values of elasticity and stiffness, we found that (1) it would be difficult to obtain the same measurement conditions in a laying position, especially in people with extreme values of this angle, and (2) the measurement of muscle parameters in a natural habitual static everyday life position is more practical and repeatable. Inclusion of men and seeking the position for examining neck muscles in the relaxed state should be the subject of future research. Moreover, we found that large percentage of variance in myofascial mechanical parameters was left unexplained, suggesting that some unknown factors contributed to the differences in stiffness and elasticity with aging. Unfortunately, we did not used other research techniques such as ultrasound imaging or magnetic resonance elastography. Therefore, we couldn’t verify if differences in fat infiltration of studied muscles^59^, or densification and fibrosis of superficial and deep fascia layers^60^ contributed to the observed variability of muscle mechanical properties.

## Conclusion

With aging, elasticity decreases and stiffness increases in the SCM and UT muscles approximately 1.5% per year between the third and ninth decade of women’ life. Aging affects mechanical properties (especially elasticity) more strongly in the SCM than UT muscle. BMI increases with aging but this weakly explains the difference in mechanical properties of the superficial neck muscles during the adult life in women. Although anterior positioning of head increases with age, it does not account for the variance in the superficial neck muscle mechanical properties with aging. Additionally, it should be underscored that the large proportion in the variance of mechanical parameters of superficial neck muscles is not explained, and thus presumably not affected by the processes directly related with aging. This suggest that other factors, such as anatomical differences in structure and composition, or habitual daily activity and loading of neck myofascial tissue, presumably account for variance in the superficial neck muscles stiffness and elasticity during sitting among women.

## Data Availability

The datasets generated during and/or analyzed during the current study are available from the corresponding author on reasonable request.
